# Highly Sensitive Sputtered ZnO:Ga Thin Films Integrated by a Simple Stencil Mask Process on Microsensor Platforms for Sub-ppm Acetaldehyde Detection

**DOI:** 10.3390/s17051055

**Published:** 2017-05-06

**Authors:** Lionel Presmanes, Yohann Thimont, Audrey Chapelle, Frédéric Blanc, Chabane Talhi, Corine Bonningue, Antoine Barnabé, Philippe Menini, Philippe Tailhades

**Affiliations:** 1 CIRIMAT, Université de Toulouse, CNRS, INPT, UPS, 118 Route de Narbonne, F-31062 Toulouse Cedex 9, France; thimont@chimie.ups-tlse.fr (Y.T.); bonning@chimie.ups-tlse.fr (C.B.); barnabe@chimie.ups-tlse.fr (A.B.); tailhades@chimie.ups-tlse.fr (P.T.); 2 LAAS-CNRS, Université de Toulouse, UPS, INSA, 7 avenue du colonel Roche, F-31031 Toulouse, France; chapelle@laas.fr (A.C.); frederic.blanc@laas.fr (F.B.); chabane.talhi@laas.fr (C.T.); Philippe.Menini@laas.fr (P.M.)

**Keywords:** gas sensors, ZnO:Ga, RF sputtering, stencil mask, metal-oxide microsensor, acetaldehyde, pulsed temperature

## Abstract

The integration of a 50-nm-thick layer of an innovative sensitive material on microsensors has been developed based on silicon micro-hotplates. In this study, integration of ZnO:Ga via radio-frequency (RF) sputtering has been successfully combined with a low cost and reliable stencil mask technique to obtain repeatable sensing layers on top of interdigitated electrodes. The variation of the resistance of this n-type Ga-doped ZnO has been measured under sub-ppm traces (500 ppb) of acetaldehyde (C_2_H_4_O). Thanks to the microheater designed into a thin membrane, the generation of very rapid temperature variations (from room temperature to 550 °C in 25 ms) is possible, and a rapid cycled pulsed-temperature operating mode can be applied to the sensor. This approach reveals a strong improvement of sensing performances with a huge sensitivity between 10 and 1000, depending on the working pulsed-temperature level.

## 1. Introduction

In 1988, Demarne et al. [1] patented the first metal-oxide semiconductor (MOS) gas sensors based on a micromachined silicon substrate. It was a groundbreaking development that has since led to mature and robust technology [2] with few examples of devices on the market, notably based on SnO_2_ and WO_3_ metal oxides. To decrease the resistivity of the gas sensitive film, as well as to improve the kinetics of the chemical reactions, commercial MOS-type gas sensors are operated in constant high temperature mode (isothermal), knowing that the interactions between the sensitive material and the surrounding gases are temperature-dependent. The most important disadvantage of MOS-type sensors is their well-known poor selectivity [3]. Functionalization of sensitive materials with suitable catalytic elements including noble metals or metal oxides can be used to improve the selectivity [4]. Recently, it has been shown that the well-defined pore structure of metal-organic frameworks was able to provide molecular sieving at the surface of ZnO nanowires [5]. Another common method to enhance selectivity is to use sensor arrays based on two or more sensing elements in order to detect gas with data of higher dimensions [6]. On the other hand, because the temperature dependence is not similar for all gases, operating a sensor at different temperatures can provide pertinent information about the gas matrix composition, or the concentration of a specific gas in a background of other gases [7]. A cycled temperature mode allowed by the low thermal mass of micro-hotplates (the thermal time constant ranges from a few to tens of milliseconds) was first introduced by Sears et al. [8] in 1989 in an attempt to avoid the interference of humidity and to enable the discrimination of several gases with a single sensor. The sensitivity can be further improved by changing the number of oxygen species at the surface of the metal-oxide when its temperature is changing. Thus, Llobet et al. [9] showed that the transient response of thermally cycled metal oxide sensors decreases the influence of humidity on sensor response and the drift in the resistance of the gas sensitive layer. In this approach, it has been shown that, with very short temperature pulses, transient sensor responses are strongly dependent on the ambient mixture of gases, so this approach can enhance sensor selectivity [10].

Because the heater operates at a relatively high temperature, the reliability of micromachined hotplates is important for MOS-type gas sensors. Since the end of the 1980s, the technology has evolved significantly and has led to performing devices at operational temperatures up to 500 °C, with a homogeneous temperature distribution over the sensing area and with minimum power consumption [2,11]. Power consumption for continuous operation is in the order of a few tens of mW, but sub-mW consumption can be reached only by using a pulsed operating temperature. These micro-hotplates can now be elaborated in an array configuration with different types of semiconducting sensitive layers and with the very interesting possibility of modulating independently of their own operating temperature [12]. Current technologies allow temperature cycling up to several millions of cycles without failure. Finally, the sensors and the near electronics can be integrated into a small substrate to obtain an autonomous embedded system.

Microsensors have many advantages as, for example, high performance, small size, low cost, and low power consumption [7]. The bibliography therefore contains many examples of microsensors onto which various sensitive layers have been deposited by different methods such as micropipetting [13,14,15,16], sputtering [17,18,19,20,21,22,23,24], precipitation–oxidation [25,26], stepwise-heating electrospinning [27], flame spray pyrolysis [28], spin coating [29], a carbo-thermal route [30], evaporation [31], metal-assisted chemical etching [32], and organic binder printing [33].

Radio-frequency (RF) sputtering is a method compatible with the industrial fabrication of miniaturized sensors by microelectronics and MEMS technologies. RF sputtering has many other advantages, such as the possibility of obtaining very thin films with nanometric scale grain sizes and very easily controlling the inter-granular porosity by varying the deposition parameters [34,35]. Films with a controlled nanostructure such as these are of great interest for their potential of acting as sensitive layers [36,37,38] and of being integrated into gas sensing devices.

In this work, we explore the use of fully compatible micromachining technologies to elaborate microheaters and deposit sensitive layers to obtain sensors at the micron scale. An elaboration of micro-hotplates was performed, and photolithographic steps and shadow masks for layer integration were investigated, with micromachining facilities at the CNRS-LAAS (Centre National de la Recherche Scientifique - Laboratoire d’Analyse et d’Architecture des Systèmes) laboratory. The sensor is based on semiconducting layers that were deposited via RF sputtering in the CIRIMAT laboratory. From many different semiconducting materials that could be deposited by this technique, an example of a very interesting one—the n-type ZnO:Ga—has been chosen in this study. Zinc oxide has received considerable attention from the scientific community for gas detection. Although ZnO is interesting because of its low cost, non-toxicity, and fast and strong response values, it can be greatly improved by doping [39]. Ga dopants have many advantages, such as the rather similar radius as compared to that of Zn, the easy substitution of Zn^2+^ by Ga^3+^ without lattice distortion, and the decrease in the resistivity of the sensor [40].

Acetaldehyde (C_2_H_4_O) is considered an air pollutant and its known to have a carcinogenic effect on humans—especially with respect to nose cancers [41]. Recent studies have highlighted the potential of pure [42,43,44,45,46,47,48,49] or doped [50,51,52] zinc oxide for the detection of this pollutant. While Ga doping has been shown to strongly improve CO sensing [53], there have hitherto been no results as to the detection of acetaldehyde using Ga-doped ZnO sensitive layers. In the present work, the objective was first to demonstrate the feasibility of the integration of a ZnO:Ga sensitive layer via a stencil mask technique and second to present its high level of sensing performances under acetaldehyde. The microsensors were tested with variable thermal sequences under a low-level concentration (0.5 ppm) of acetaldehyde.

## 2. Experimental

Thin sensitive films were deposited with an Alcatel SCM 400 (Alcatel, France) apparatus using a homemade sintered ceramic target of pure ZnO:Ga with a relative density around 70% (9 cm in diameter). The RF power was lowered at 50 W to avoid target reduction [54], and the pressure inside the chamber was lower than 2 × 10^−5^ Pa before deposition. During the deposition of the films, the target-to-substrate distance was fixed at 7 cm (Table 1). The thicknesses of deposited thin films have been set to 50 nm on microsensors and 100 nm on glass substrates for structural characterizations. A pressure of 2 Pa was set to promote the intergranular porosity [55].

Film thicknesses were measured using a Dektak 3030ST stylus profilometer across a step obtained by the lift-off of a felt pen line in acetone after deposition. The structural properties were determined by X-ray diffraction (XRD) using a Siemens D4 diffractometer with the Cu K_α_ radiation (K_α_ = 1.5418 Å). Microscopic studies were realized with a Veeco Dimension 3000 atomic force microscope (AFM) equipped with a super sharp TESP-SS Nanoworld tip (nominal resonance frequency 320 KHz, nominal radius curvature 2 nm).

For sensing measurements, sensors were placed into a chamber with an alternating flow of air and 0.5 ppm of acetaldehyde. The composition and humidity of the gas mixture were controlled via mass flow controllers (MFCs). The heating and the sensing resistors of each sensor were connected to a source measurement unit (SMU). The entire test bench was automatically controllable thanks to a suitable and compatible interface and dedicated software. After a period of stabilization of 2 h under synthetic air, 0.5 ppm of acetaldehyde was introduced 5 times for 15 min with return periods in air of 30 min between each exposure to the target gas. The global flow (200 sscm) and the relative humidity (50%) remained constant during both air and target gas sequences. The response of gas sensor was calculated according to the formula: S = R_gas_/R_air_ (where R_air_ and R_gas_ are the resistance in air and 0.5 ppm of C_2_H_4_O, respectively).

## 3. Preparation of Microheaters

**Figure 1:**
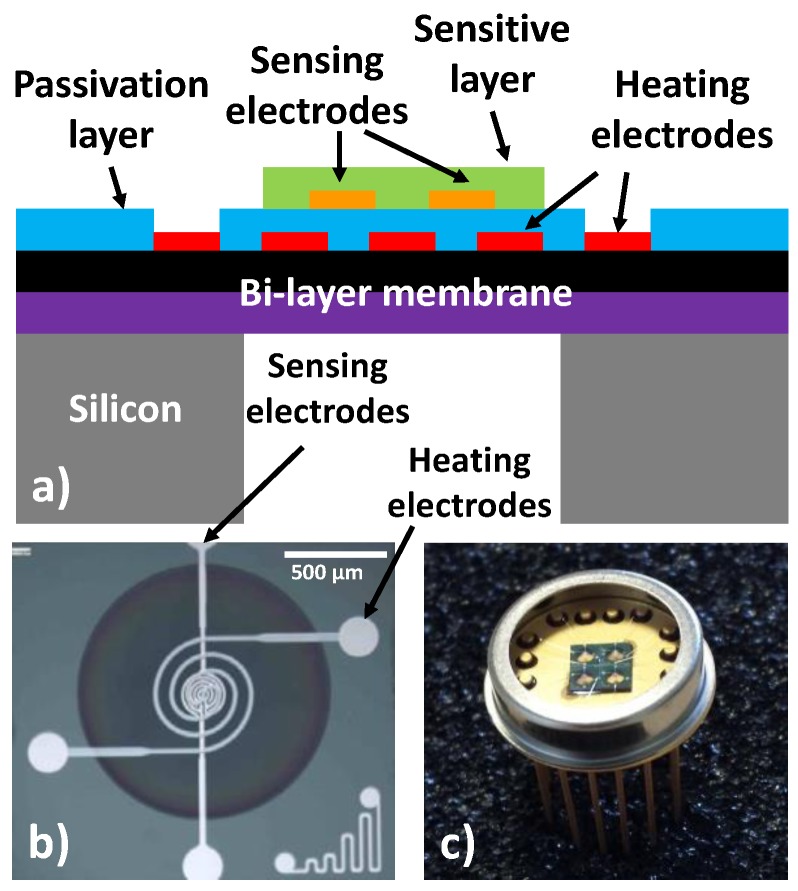
Micro-hotplate gas sensor: (**a**) a cross-sectional schematic view; (**b**) a chip top view; (**c**) the multi-sensor (4 chips) packaged on a TO-9 support.

The tested devices were developed on optimized micro-hotplates that can work at high temperatures and low power consumption (500 °C; ~55 mW) with a very good stability and reproducibility. These silicon structures were elaborated using standard photolithographic processes. In order to avoid edge effects and to improve thermo-mechanical behavior, a circular membrane and heater geometry (Figure 1) were chosen. The design was elaborated to optimize temperature homogeneity in the center of the heated area onto which the sensing electrodes were deposited.

The platform consists of a silicon bulk on which a thermally resistive bilayer SiO_2_/SiN_x_ membrane was grown. Afterwards, Pt metallization was realized via lift-off to define a heating resistor and the sensing electrodes. Contacts were opened in a previously deposited passivation layer. Finally, the rear side of the bulk was etched to release the membrane in order to increase the thermal resistance and then to limit thermal dissipation. Figure 1b shows the top view of the final membrane. This technology can prepare multi-sensors on which more than one sensing chip can be obtained in the same device (a 4-chip sensor is presented as an example in Figure 1c). This type of multi-sensor is especially suitable for operation in complex atmospheres containing various interfering gases and obtaining a good selectivity.

**Figure 2:**
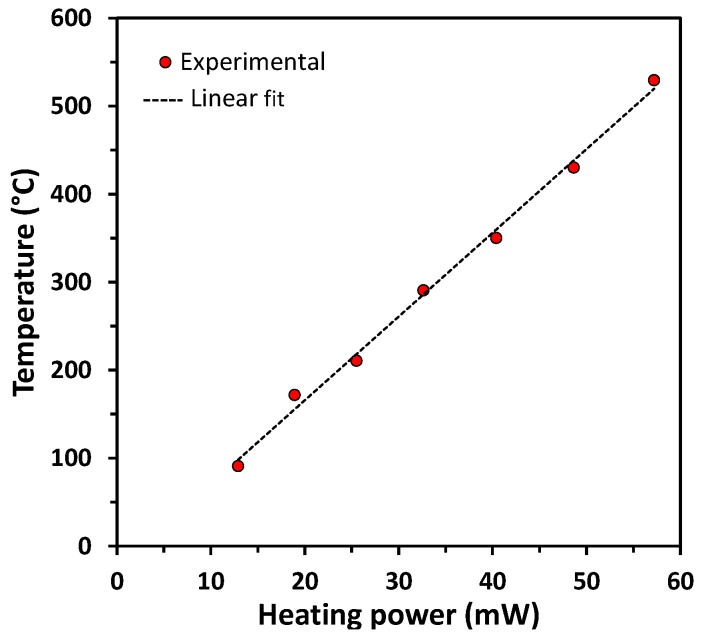
Temperature reached in the center of the microheater vs. the applied heating power.

Thermal measurement of the micro-hotplate surface with an IR camera allowed the calibration between the power applied and the resulting heating temperature of the membrane. The results given in Figure 2 show a good linear relation between the power applied and the temperature measured. The heating platform makes it possible to heat from room temperature to 550 °C in 25 ms, and the cooling time is of the same order of magnitude. This type of platform can thus generate very rapid temperature variations, which is suitable for operating the sensor in a pulsed mode. At the end of the process (before dicing the chips), it is possible to locally deposit a metal-oxide layer onto the electrodes to form the sensing thin film resistor. This will be described in the paragraph below, which is dedicated to the integration of ZnO:Ga by using a deposition through a shadow mask.

## 4. Integration of N-type ZnO:Ga by the Shadow Mask Process

### 4.1. Characterization of the ZnO:Ga Layer

**Figure 3:**
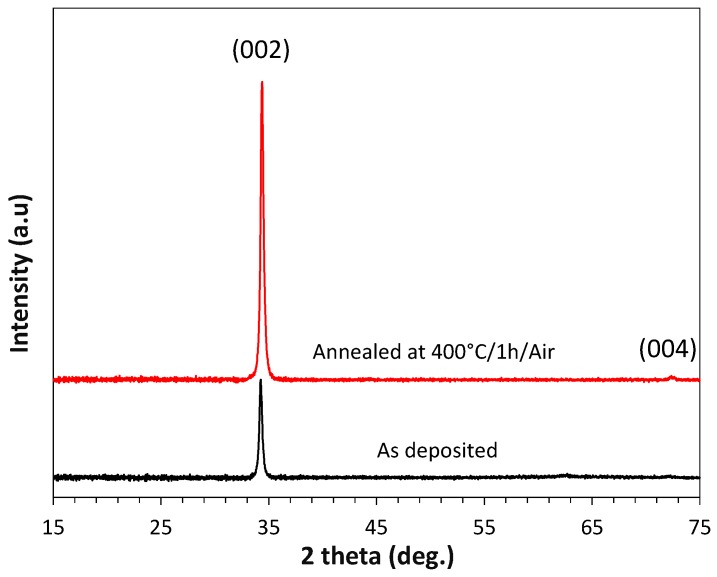
XRD pattern of a 100 nm ZnO:Ga film deposited on glass, before and after annealing under air at 400 °C.

For structural characterization, 100-nm-thick films have been deposited on glass substrates in the condition defined in Table 1. The XRD patterns acquired at room temperature for as-deposited and annealed ZnO:Ga thin films have been reported in Figure 3. The results confirm that zinc oxide is crystallized (space group P6_3_mc with lattice parameters a = 3.35(1) Å and c = 5.22(6) Å according to L. Weber [56]) with a single growth orientation along the (001) direction, which is largely reported in the bibliography for ZnO thin films deposited by physical vapor deposition techniques [57,58]. The lattice parameter c calculated from the (002) peak using a pseudo-voigt function is equal to 5.23(0) Å for the as-deposited thin film and becomes equal to 5.21(3) Å after a 400 °C annealing treatment under air for 4 h. The decrease of the c parameter after annealing could be due to a possible zinc substitution by silicon from the substrate [59], the removal of a lattice disorder, and the effect of the film/substrate interface strength due to the difference of the thermal expansion coefficients [60]. We noticed an increase in the (002) peak intensities after air annealing, which might be explained by the increase in the ZnO crystallized fraction caused by the annealing process.

The Scherrer relation [61] is defined by Equation (1):

(1) $$d=\frac{K\times \lambda }{FWHM_{sample}\times \cos\theta }$$

where *d* is the size of the crystallites, *K* a shape correction factor, *λ* the X-ray wavelength, *FWHM_sample_* the width of the peak at its half maximum amplitude (corrected from instrumental contribution), and *θ* the peak position. The widths of the (002) peaks were calculated from a pseudo-voigt function after removing copper Kα_2_ using EVA software. Considering isotropic shape crystallites, where *K* is equal to 0.9, and by neglecting the possible micro-strain component, the crystallites sizes were estimated. The average crystallites sizes of the 100 nm as-deposited ZnO:Ga thin film were about 33 nm. Thermal treatment increased the mean crystallite size by 5 nm, and the behavior is in agreement with the literature [62,63].

**Figure 4:**
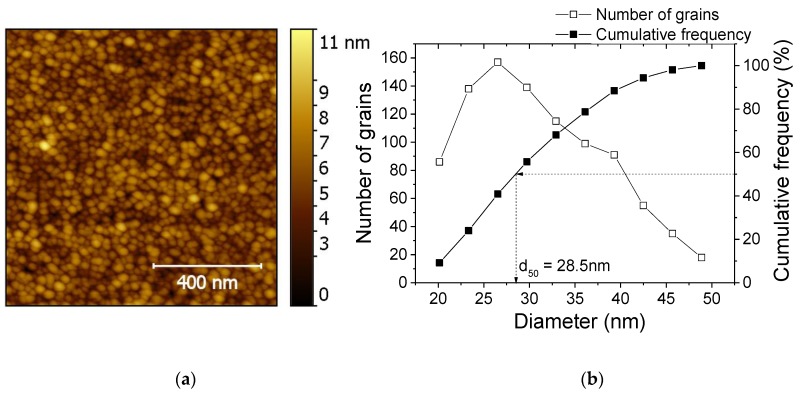
(**a**) AFM image of a 50-nm-thick ZnO:Ga film annealed at 400 °C for 1 h under an air atmosphere. (**b**) Grain size distribution deduced from the image analysis.

In the sensor device, thinner zinc oxide layers were deposited to attain better sensitivity. As a result, the observation of the surface of the ZnO:Ga layer by AFM has been made on films deposited with a thickness of 50 nm (after annealing). The AFM image reported in Figure 4a shows a classical grain morphology, which consists of surface domes (top of the grown column). This characteristic is often mentioned in the literature [64,65]. The in-plane average grain size distribution (Figure 4b) was estimated by an immersion threshold thanks to the Gwyddion software [66].

The average grain size determined as the diameter of 50% of the total cumulative frequency (d_50_) is 28 nm for the 50-nm-thick film annealed at 400 °C, and the maximum peak-to-valley amplitude was found to be equal to 11 nm. In the case of the 100-nm-thick films (used for the XRD analysis), the surface morphology (not exposed here) was similar to the 50-nm-thick films, except that the median grain size was larger (60 nm).

### 4.2. Description of the Integration Process via Stencil Mask

**Figure 5:**
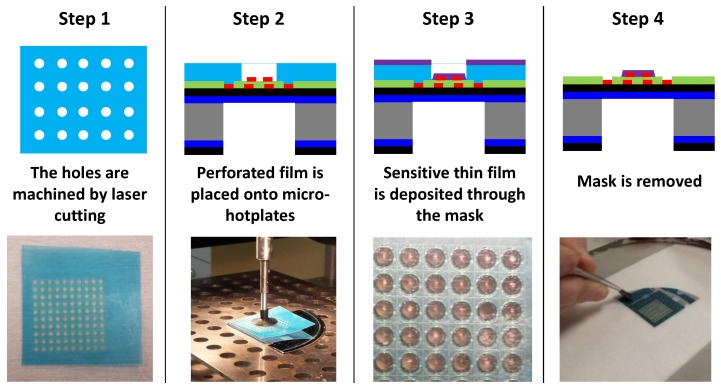
Main steps in the integration process of ZnO:Ga sensitive layers using a shadow mask.

**Figure 6:**
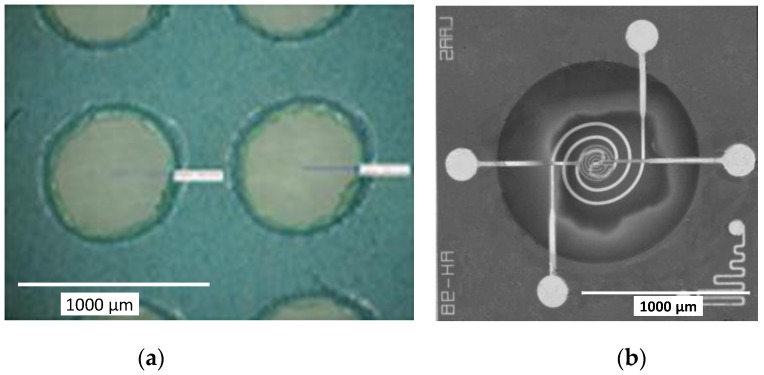
Optical microscopy image of (**a**) the stencil mask (hole diameter: 600 µm) and (**b**) the ZnO:Ga layer deposited onto the electrode area after the removal of the stencil mask.

The main disadvantage of the lift-off technique is the complexity of the various and necessary steps, which involve expensive equipment. Moreover, during the deposition of the photoresist, its development, and the removal of the remaining resist mask, the interaction of the solutions used with the sensitive layer can lead its dissolution and/or contamination. These are the reasons why the possibility of depositing the layer through a stencil mask has been evaluated. The entire process is shown in Figure 5. The mask has been opened in an adhesive film made of polyvinyl chloride with a thickness of 75 µm. The holes made with a simple cutting laser machine had a diameter close to 600 µm. The mask diameter was chosen to be lower than that of the membrane (1.2 mm) but higher than the active area (400 µm) where the interdigitated sensing electrodes are located. The mask with 100 cut out holes (Figure 5, Step 1) was aligned and stuck onto the surface of a quarter of a micro-machined silicon wafer (with 100 sensing chips). To achieve mask placement with high accuracy, this step was performed with an optical microscope and a manual pick-and-place machine (Figure 5, Step 2). After vacuum deposition of the 50-nm-thick ZnO:Ga sensitive layer via RF sputtering (Step 3), the stencil mask was simply peeled off after a 120 °C thermal treatment over a few tens of seconds (Step 4). Figure 6 shows the resulting ZnO:Ga layer, which is located on top of the membrane and, above all, well covering the sensing electrode area.

## 5. Sensing Tests

**Figure 7:**
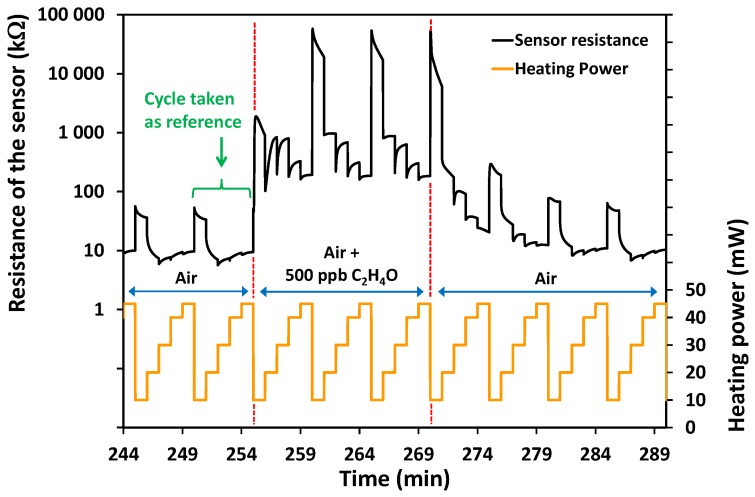
Variation of the resistance of the sensing layer with the heating power and the gas composition.

In this first study, the microsensor based on ZnO:Ga semiconducting layers have only been tested under sub-ppm concentrations (500 ppb) of acetaldehyde (C_2_H_4_O). Five levels of power were applied to the heater to explore the sensing performances from 10 to 45 mW. The sensor was held at each heating step for 1 min, and every 5 min, this basic cycle was repeated. During the sensing test, a dissymmetrical procedure has been used: Three cycles were repeated under target gas (acetaldehyde), while 6 cycles were used in air to ensure total recovery of the signal. The target gas was introduced 5 times throughout the experiment to test the repeatability. Figure 7 shows the last 2 of the 6 previous cycles under air, the three cycles under 500 ppb of acetaldehyde, and the first 4 of the following 6 cycles under air.

We observed that, before the introduction of C_2_H_4_O, the evolution of the resistance during the power ramp was stable from one cycle to another. On the other hand, when the atmosphere switched from air to 500 ppb of acetaldehyde, the sensing resistance was substantially shifted to higher values. The stabilization of the resistance was typically obtained from the second cycle. When the atmosphere was switched again to pure air, recovery was achieved after the third power ramp cycle. The cycle obtained in the fourth cycle was similar to the last cycle under air before introducing acetaldehyde.

**Figure 8:**
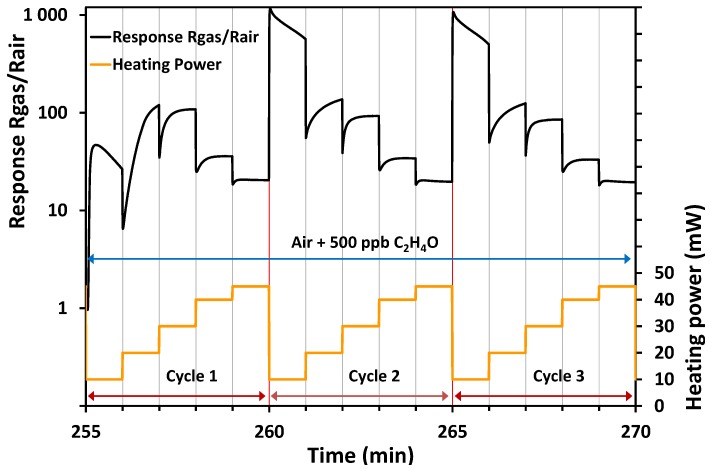
Response of the sensor (R_gas_/R_air_) under 500 ppb of acetaldehyde in a temperature-cycled mode.

The estimation of the gain of resistance was difficult to obtain directly from the variation of the signal reported in Figure 7. This is the reason why the response R_gas_/R_air_ was presented in Figure 8. As the resistance of the sensitive layer was constantly changing with the value of the power and during each power step, it was therefore not possible to take a fixed value as a reference. The entire variation of the resistance during the last power ramp cycle before gas introduction was then taken as a reference (Figure 7). In Figure 8, for each cycle under acetaldehyde, the values of the resistance are divided by the values of the last cycle under air. From these curves, the very high response of the ZnO:Ga sensitive layer is highlighted. The best values were obtained from the lowest power heating, even though at 10 and 20 mW the stabilization could never be reached, while at 30, 40, and 45 mW the stabilization could be obtained after approximately 30 s. Regardless of the power applied, the signal was significant even at the highest power value of 45 mW for which the response R_gas_/R_air_ remains around 20. It is difficult to compare the present results with those of the other authors because, to our knowledge, nobody has yet published a study concerning the sensitivity of ZnO:Ga toward acetaldehyde. However, the bibliography confirms that doping, for instance, with Co [50,51], Cu [50], or Ru [51], improves the sensing performance of ZnO toward acetaldehyde in comparison with undoped ZnO.

For now, it is not yet known if heating cyclically to high power values (40 and 45 mW for instance) has a significant effect. This is the reason why more experiments and investigations are in progress. Actual power cycles will be compared to other measurement modes as a constant temperature mode and a cycled mode with a high-temperature baseline. Moreover, it is necessary to corroborate the current results with complementary experiments under various acetaldehyde concentrations.

## 6. Conclusions

Micro-hotplates were first prepared using silicon microtechnologies. Because the microheater was designed for use on a thin membrane, it was possible for us to generate very fast temperature variations (from room temperature to 550 °C in 25 ms), and a rapid temperature cycled mode could be applied. A method using a stencil mask was developed so that the sensitive layer can avoid contact with the products used during the photolithography steps. This process was successfully tested during the integration of the ZnO:Ga sensitive layer. The variation of the resistance of this 50-nm-thick sensitive layer was measured under 500 ppb of acetaldehyde. The very high response obtained was between 10 and 1000, depending on the working temperature. Using a rapid temperature cycled mode is a good opportunity to evaluate the selectivity of the sensor in other interfering gases, and such a study will be carried out in a next step.

## Figures and Tables

**Table 1:** Deposition parameters of thin sensitive films.

Target Material	ZnO:Ga
Magnetron	Yes
Substrates	Glass and Micro-Hotplate
Power	50 W
Argon pressure	2 Pa
Target to substrate distance	7 cm
Deposition rate	2.3 nm/min
